# Helsinki Stroke Model Is Transferrable With “Real-World” Resources and Reduced Stroke Thrombolysis Delay to 34 min in Christchurch

**DOI:** 10.3389/fneur.2018.00290

**Published:** 2018-04-30

**Authors:** Teddy Y. Wu, Erin Coleman, Sarah L. Wright, Deborah F. Mason, Jon Reimers, Roderick Duncan, Mary Griffiths, Michael Hurrell, David Dixon, James Weaver, Atte Meretoja, John N. Fink

**Affiliations:** ^1^Department of Neurology, Christchurch Hospital, Christchurch, New Zealand; ^2^Department of Radiology, Christchurch Hospital, Christchurch, New Zealand; ^3^Department of Emergency Medicine, Christchurch Hospital, Christchurch, New Zealand; ^4^Department of Neurology, Helsinki University Hospital, Helsinki, Finland

**Keywords:** thrombolysis, door-to-needle, delay, resource, stroke

## Abstract

**Background:**

Christchurch hospital is a tertiary hospital in New Zealand supported by five general neurologists with after-hours services provided mainly by onsite non-neurology medical residents. We assessed the transferrability and impact of the Helsinki Stroke model on stroke thrombolysis door-to-needle time (DNT) in Christchurch hospital.

**Methods:**

Key components of the Helsinki Stroke model were implemented first in 2015 with introduction of patient pre-notification and thrombolysis by the computed tomography (CT) suite, followed by implementation of direct transfer to CT on ambulance stretcher in May 2017. Data from the prospective thrombolysis registry which began in 2012 were analyzed for the impact of these interventions on median DNT.

**Results:**

Between May and December 2017, 46 patients were treated with alteplase, 25 (54%) patients were treated in-hours (08:00–17:00 non-public holiday weekdays) and 21 (46%) patients were treated after-hours. The in-hours, after-hours, and overall median (interquartile range) DNTs were 34 (28–43), 47 (38–60), and 40 (30–51) minutes. The corresponding times in 2012–2014 prior to interventions were 87 (68–106), 86 (72–116), and 87 (71–112) minutes, representing median DNT reduction of 53, 39, and 47 minutes, respectively (*p*-values <0.01). The interventions also resulted in significant reductions in the overall median door-to-CT time (from 49 to 19 min), CT-to-needle time (32 to 20 min) and onset-to-needle time (168 to 120 min).

**Conclusion:**

The Helsinki stroke model is transferrable with real-world resources and reduced stroke DNT in Christchurch by over 50%.

## Introduction

Intravenous thrombolysis with tissue plasminogen activator (tPA)—alteplase—is the current standard treatment for ischemic stroke within 4.5 h of symptom onset (1). The benefit of tPA diminishes with treatment delay (1, 2); every minute delay to treatment is associated with nearly two disability free health days lost (2). Successful reduction in stroke treatment times through implementing systematic thrombolysis protocol has been reported in stroke centers around the world (3–5) with the most rapid stroke treatment reported to-date achieved in Helsinki (6). The Helsinki stroke model consisted of 12 interventions aimed at reducing treatment delays prior to thrombolysis and the adaptation of this model resulted in 18-min reduction in thrombolysis delay to 25 min in Melbourne within 4 months (7). The American Heart Association/American Stroke Association Target: Stroke initiative also recommended a similar 10-step intervention (8) aimed at reducing door-to-needle times (DNT) and resulted in nearly 80% relative increase in the proportion of stroke tPA administered within 60 min of hospital arrival (9).

The ethos of these models focuses on patient pre-notification, rapid clinical assessment, image acquisition, and treatment administration in a chain of responses requiring parallel processing by on-site radiology and neurology personnel. This is possible in healthcare settings with access to 24/7 on-site specialist personnel, which is not available in hospitals throughout New Zealand. The transferrability of thrombolysis models such as the Helsinki model in this setting is uncertain. The aim of this paper is to report the transferrability and impact on DNT of the Helsinki stroke thrombolysis model to a New Zealand tertiary hospital.

## Materials and Methods

### The Healthcare Setting

Christchurch Hospital is a government funded tertiary hospital located in Christchurch city in the South Island of New Zealand. It provides hospital care to the Canterbury region with a catchment population of ~500,000 and is the second largest public hospital by catchment population in New Zealand. Christchurch Hospital admits approximately 600 ischemic stroke patients annually to a dedicated 15-bed acute stroke unit and is the only hospital in the South Island with capacity for endovascular clot retrieval. Most stroke patients are ultimately cared for by the stroke rehabilitation physician in the stroke unit, while stroke patients considered for thrombolysis or endovascular clot retrieval are admitted under the neurology bed card within the stroke unit for the first 24–48 h. The neurology team consists of five general neurologists with two neurology trainee residents and one rotating medical resident. The residents participate in a 1 in 7 after-hours on-call roster (16:00–22:30) with residents from nephrology and infectious diseases. The night roster (22:30–08:00) is shared by also the medical specialty residents together with rotating internal medicine residents. The after-hours on call resident is responsible for clinical management of neurology, nephrology, and infectious diseases patients. The neurologists share a 1 in 4 on-call roster and are available by phone 24/7 and are generally on site until 17:00 and for this reason we consider business hours to be between 08:00 and 17:00 on non-public holiday weekdays. The after-hours stroke cover is provided mainly by non-neurology residents and because of this, a 4-monthly stroke pathway presentation has been held to coincide with changeover of rotating residents. Further individual feedback sessions are available. Additionally, there is an ongoing annual education and update on the acute stroke service to the St. John Ambulance paramedics.

### The Thrombolysis Registry and Institutional Approval

A prospective stroke thrombolysis registry was started in 2012. The registry contains basic demographic and clinical information for patients receiving tPA. National Institutes of Health Stroke Scale (NIHSS) was available in most patients and retrospectively calculated for patients missing this variable. The registry collected a number of time metrics, including symptom onset time, hospital arrival time, time of imaging, and thrombolysis. For inpatient strokes, the time stroke page was initiated and was used as the arrival time. DNT was taken as the time taken in minutes from recorded arrival time at the emergency department (ED) to the recorded time of tPA bolus administration. Door to computed tomography (CT) time was defined as time taken in minutes from arrival time in ED to completion of non-contrast CT images. Stroke mimics who were thrombolysed were also included in the present analysis. Symptomatic intracerebral hemorrhage was defined as per Safe Implementation of Thrombolysis in Stroke-Monitoring Study (SITS-MOST) (10). Long-term outcome data were unavailable as clinical follow up was not routine.

For the present study, we included all consecutive patient receiving tPA for presumed acute ischemic stroke between January 2012 and December 2017 even if the final diagnosis was a stroke mimic. We excluded two inpatient strokes with missing stroke page time.

The thrombolysis registry forms part of the Christchurch Stroke registry study and the prospective National New Zealand Thrombolysis Registry as mandated by the Ministry of Health and has approval from the national Health Disability Ethics Committee and Christchurch Hospital research office. As this is a quality improvement, observational registry without deviation from routine care or additional patient contact, consent for inclusion into this analysis was not required by the New Zealand research legislation, allowing for inclusion of all consecutive patients.

### Treatment Criteria

Acute stroke thrombolysis was introduced in Christchurch hospital in April 2002 for patients presenting within 3 h of symptoms onset or from last known well time. The treatment time window was extended to 4.5 h in 2009 following publication of the third European Cooperative Acute Stroke Study (ECASS III) (11). We have no age restriction but restricted tPA for patients with good pre-morbid functional status (pre-stroke modified Rankin Scale score 0–3). There is no NIHSS restriction but treatment is generally not offered to patients with minor strokes symptoms with NIHSS ≤4 with the exception of isolated homonymous hemianopia. In patients on warfarin, tPA is given to patients with an international normalized ratio ≤1.5. In patients known to be on dabigatran, idarucizumab is given prior to tPA. Other standard contraindications apply as per best practice guidelines (12). Informed consent was provided verbally by the patient or next of kin. In the situation where a patient could not consent and the next of kin could not be contacted, a medical decision would be made by the neurologist to proceed with treatment.

### Christchurch Hospital Stroke Thrombolysis Model

#### Previous Acute Stroke Model

Prior to 2015, acute stroke patients were selectively pre-notified to ED triage by the St. John Ambulance paramedic if a significant deficit was evident. However, the acute stroke team was not notified until after ED physician assessment. The stroke team then assessed the patient in ED prior to requesting CT scan, which is the imaging modality of choice for acute stroke at our hospital. The CT suite is located on the first floor of the hospital and the stroke patient was not routinely given an immediate priority for imaging. Following imaging if the patient was deemed eligible for thrombolysis the patient was transferred to the acute stroke unit located also on the first floor, where the tPA was administered.

#### New Acute Stroke Model

Due to median DNT of more than 60 min even during business hours, an effort was made to adopt the key components of the Helsinki Stroke Thrombolysis model (6). In January 2015, a “Code Stroke” pre-notification pathway was introduced where a page was made to the acute stroke team initiated by ED triage upon paramedic notification of an incoming stroke patient. The page was also received by the CT radiographer, who was encouraged to stop processing further non-urgent CT requests until after the imaging of stroke patient had completed. The stroke team assessed the patient in the ED cubicle to determine if the patient had a treatable neurological deficit. If the patient had a treatable deficit, a scan request was sent and patient was taken for CT scanning. Because of resource constraints and in agreement with radiology, only patients deemed suitable for intervention (thrombolysis or endovascular clot retrieval) would proceed with immediate CT imaging. Imaging was immediately interpreted by the acute stroke team in consultation with onsite radiologist or radiology resident. Although multimodal imaging with CT angiography and perfusion was available, it was not performed in most patients under the old stroke model and the decision to administer alteplase was made after non-contrast imaging. In early 2016, CT perfusion and angiography were added to the acute stroke imaging protocol and thrombolysis was usually initiated after completion of all advanced imaging. Thrombolysis is administered in a clinical cubicle within the radiology suite although bolus administration on the CT table prior to advanced imaging is encouraged.

In May 2017 following consultation and agreement with ED, St. John Ambulance and radiology, the final step in the new thrombolysis model was introduced. The main change was for patient to be assessed on the ambulance stretcher in an ED bed cubicle immediately after arrival and if deemed a thrombolysis candidate the patient is transferred directly to CT on the ambulance stretcher.

The St. John Ambulance paramedics are encouraged to insert an 18-gauge intravenous line in the antecubital fossa prior to arrival. Blood testing was performed in all patients but contrast imaging and tPA would proceed without waiting for laboratory results in most cases. In patients anticoagulated with warfarin, point-of-care international normalized ratio testing was available. In patients known to be on dabigatran, our departmental policy is to administer the reversal agent idarucizumab in the CT suite prior to thrombolysis without waiting for coagulation laboratory results to avoid treatment delay. Idarucizumab is kept in a drug room within ED and can be taken with patient to the CT suite or retrieved from ED by the stroke nurse once a decision to thrombolyse has been made. The “Code Stroke” pathway is only available between 08:00 and 22:30 as immediate CT during overnight is not available due to the CT radiographers operating on a call-back roster system. Table 1 summarizes the key components of the current Christchurch Hospital stroke thrombolysis model.

**Table 1:** The new stroke thrombolysis model in Christchurch Hospital.

	Christchurch thrombolysis model—modified from the Helsinki Stroke model (6)
Stroke patient pre-notification	St. John Ambulance paramedic pre-notifies ED triage room with patient clinical and demographic details including national hospital index number unique to individual patient. An estimated time of arrival is given. CT radiographer, neurology resident, and stroke nurse are paged *via* “Code Stroke” call to switchboard

Medical history	Patient electronic medical record, including general practitioner, next of kin details, laboratory results, and a South Island wide PACS system is examined. Next of kin contacted for collateral history prior to arrival as required

Direct to CT	Upon arrival, the patient is examined on the ambulance trolley to determine eligibility for thrombolysis. If deemed, eligible patient is transported to the CT suite located on first floor of the hospital. Electronic ordering of CT is performed by stroke nurse or ED physician

Intravenous line/laboratory testing	Patients usually have 18-gauge antecubital fossa intravenous line, otherwise this is inserted on arrival in ED. Bloods drawn on arrival or on CT table. Blood results not required prior to contrast CT or thrombolysis

Point-of-care INR/administration of idarucizumab	Point-of-care INR available. Idarucizumab stored in ED fridge and taken with patient to CT suite and administered there prior to thrombolysis

tPA in CT suite	Bolus given on table, but usually in a clinical cubical adjacent to the CT scanner

Regular paramedic/resident education	Four monthly stroke model education session with rotating registrars. Annual formal paramedic education and update on stroke statistics

*CT, computed tomography; ED, emergency department; INR, international normalized ratio; PACS, picture archiving and communication system; tPA, tissue plasminogen activator*.

### Statistical Analysis

We analyzed the impact of the change to the stroke thrombolysis model by comparing time metrics achieved in the three epochs during the staged introduction of the new thrombolysis model—(1) the pre-intervention period (2012–2014), (2) following introduction of code stroke pre-notification system (2015–2017 April), and (3) full implementation of new stroke model following introduction of “direct to CT” transfer (May–December 2017). We reported for each epoch the overall, in-hours (08:00–17:00 non-public holiday weekdays) and after-hours time metrics. We analyzed the change in treatment and imaging time metrics between pre-interventional period and following the full implementation of the new stroke model. We included patients treated overnight (22:30–08:00), where the acute stroke model was non-operational, in the after-hours statistics. The annualized overall and in-hours DNT during the study period were plotted graphically to illustrate the changing trend overtime. Additionally, the impact of advanced imaging and stroke severity on the overall DNT over the years was also analyzed.

Where appropriate, all data pertaining to the NIHSS, age, and treatment delays were presented as median with interquartile range, grouped by year to demonstrate trends in time. Comparison of groups was analyzed using independent samples Mann–Whitney *U* or Kruskal–Wallis tests. A two-sided *p*-value <0.05 was considered statistically. Analyses were performed using IBM SPSS 23 software (IBM Corp., Armonk, NY, USA).

## Results

### Patient Characteristics and Treatment Numbers

There was a progressive increase in the number of thrombolysed stroke patients each year (Table 2) and in the 2017 calendar year 67 patients were given tPA representing a threefold increase compared to 2012. A total of 255 patients were included in the analysis. Over time there were no significant differences in the age of thrombolysed patient (*p* = 0.278) with a trend toward lower NIHSS (*p* = 0.052). The use of advanced imaging also increased over the years and was almost routine in 2017, performed in 91% of tPA patients. Idarucizumab was given to reverse the anticoagulant effects of dabigatran prior to thrombolysis in seven (10.4%) patients in 2017 with median DNT of 40 min.

**Table 2:** Baseline characteristics, time to imaging, use of multimodal imaging, and idarucizumab in thrombolysed patients at Christchurch Hospital.

	Total	Age	National Institutes of Health Stroke Scale	Door-to-imaging (minutes)	Multimodal imaging	Dabigatran reversal
2012	23	72 (65–81)	16 (9–21)	48 (40–57)	1 (4%)	0
2013	39	73 (58–82)	13 (8–20)	48 (40–89)	2 (5%)	0
2014	28	80 (64–87)	17 (11–21)	53 (43–64)	1 (4%)	0
2015	43	73 (60–81)	8 (5–18)	46 (36–57)	6 (14%)	2 (4.7%)
2016	55	73 (61–81)	11 (6–20)	30 (24–42)	32 (58%)	4 (7.3%)
2017	67	75 (67–83)	10 (5–17)	20 (15–27)	61 (91%)	7 (10.4%)

Total	255	74 (62–82)	13 (7–20)	39 (24–53)	103 (40%)	13 (5%)

Symptomatic intracerebral hemorrhage occurred in 10 (3.9%) patients during the study period, with 1 (2.1%) case of SICH observed in the 8 months between May and December 2017 following full implementation of the new stroke model. A stroke mimic was diagnosed in 8 (3.1%) patients (five with non-organic functional symptoms, two with migraine, and one related to epileptic seizure). The stroke mimic treatment rate in patients without prior multimodal CT was 4.6% (7/152) compared to 1.0% (1/103) with pre-treatment advanced imaging.

### Reduction in Treatment Metrics Over Time

**Figure 1:**
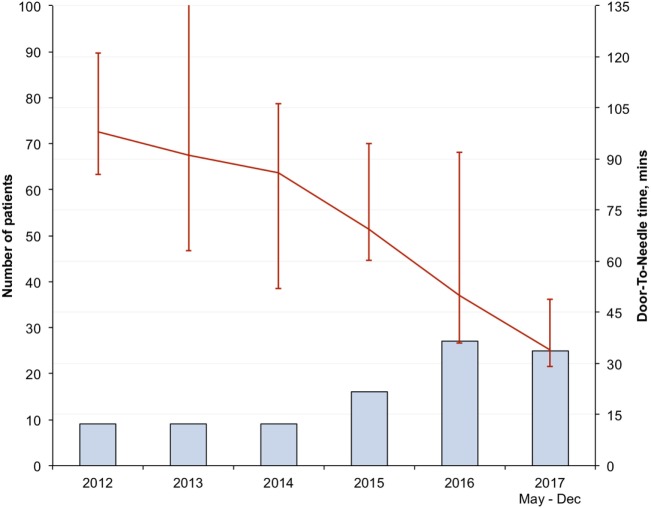
In-hours median door-to-needle time in minutes.

**Figure 2:**
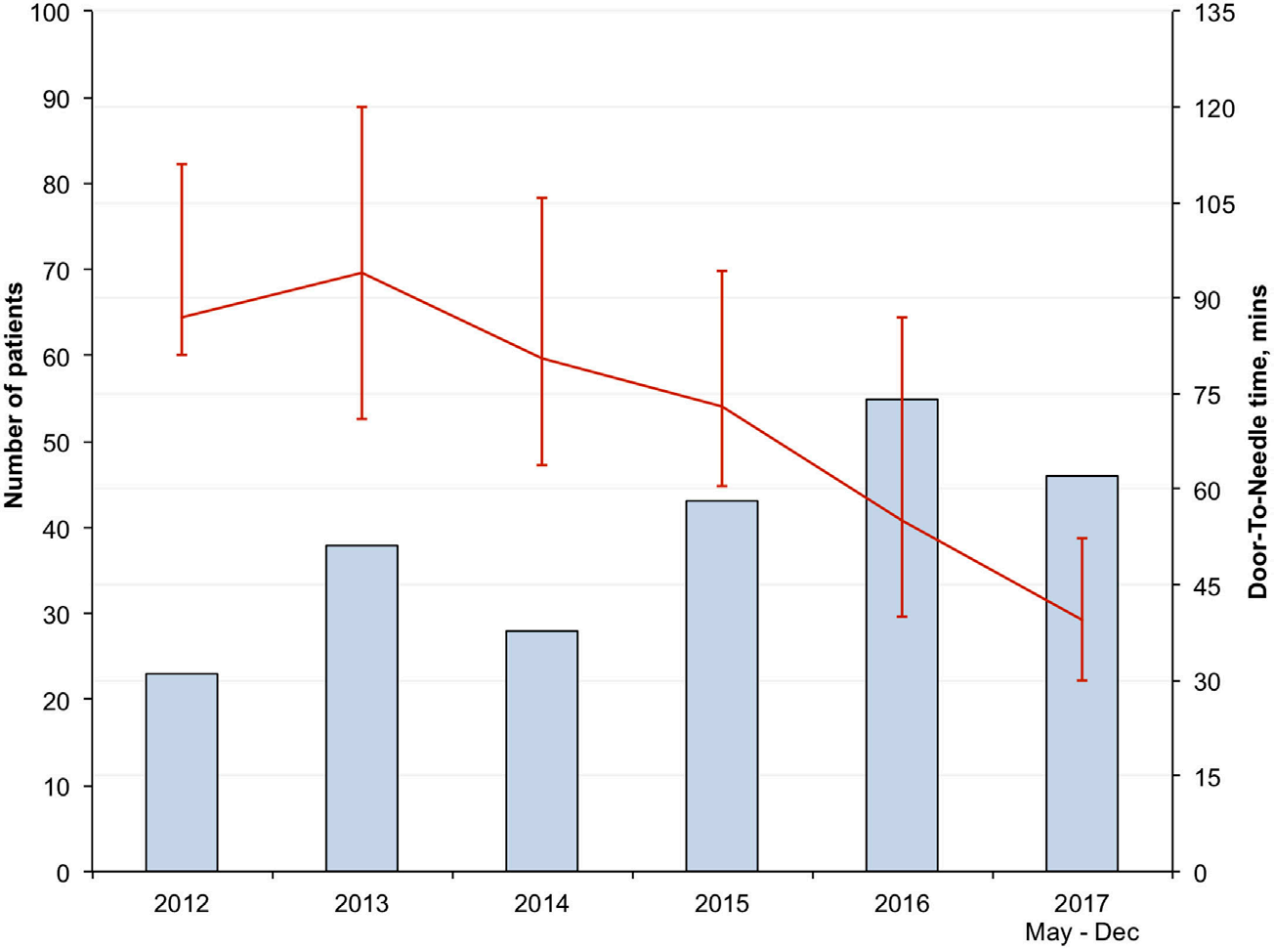
Overall median door-to-needle time in minutes.

There was progressive reduction in DNT, since 2012 with the largest reduction seen in the in-hours DNT from 87 (68–106) minutes in the pre-intervention period to 34 (28–43) minutes after full implementation of the new stroke model (*p* < 0.01) (Figure 1; Table 3). The overall median DNT time also reduced from 87 (71–112) minutes to 40 (30–51) minutes (Figure 2; Table 3), while the after-hours DNT went down to 47 (38–60) minutes from 86 (72–116) minutes (*p* < 0.01). The overnight median DNT was relatively unchanged from 87 min in the pre-intervention period to 78 min in with the new stroke model. The proportion of patients treated within 60 min of hospital arrival also increased from 12% in the pre-intervention period to 79% in 2017.

**Table 3:** Summary of different time metrics represented in minutes [median (interquartile range)] for thrombolysed patients at Christchurch Hospital.

	Pre-intervention period 2012–2014	Stroke pre-notification 2015–2017 April	Full stroke model 2017 May–December	*p-*Value
**In-hours**				
Door-to-needle	87 (68–106)	61 (41–83)	34 (28–43)	<0.01
Door-to-computed tomography (CT)	48 (38–67)	35 (24–47)	17 (11–20)	<0.01
CT-to-needle	36 (16–49)	19 (13–33)	18 (10–24)	<0.01
Onset-to-needle	160 (125–195)	122 (96–162)	101 (80–140)	<0.01

**After-hours**				
Door-to-needle	86 (72–116)	65 (52–84)	47 (38–60)	<0.01
Door-to-CT	50 (41–65)	35 (25–50)	22 (15–32)	<0.01
CT-to-needle	31 (20–50)	28 (19–41)	24 (17–33)	0.24
Onset-to-needle	170 (147–195)	135 (115–160)	123 (105–186)	<0.01

**Overall**				
Door-to-needle	87 (71–112)	63 (48–84)	40 (30–51)	<0.01
Door-to-CT	49 (40–65)	35 (24–48)	19 (11–24)	<0.01
CT-to-needle	32 (20–49)	26 (15–37)	20 (14–31)	<0.01
Onset-to-needle	168 (145–195)	130 (107–160)	120 (87–155)	<0.01

### Impact of Advanced Imaging and Stroke Severity on Treatment Time

**Figure 3:**
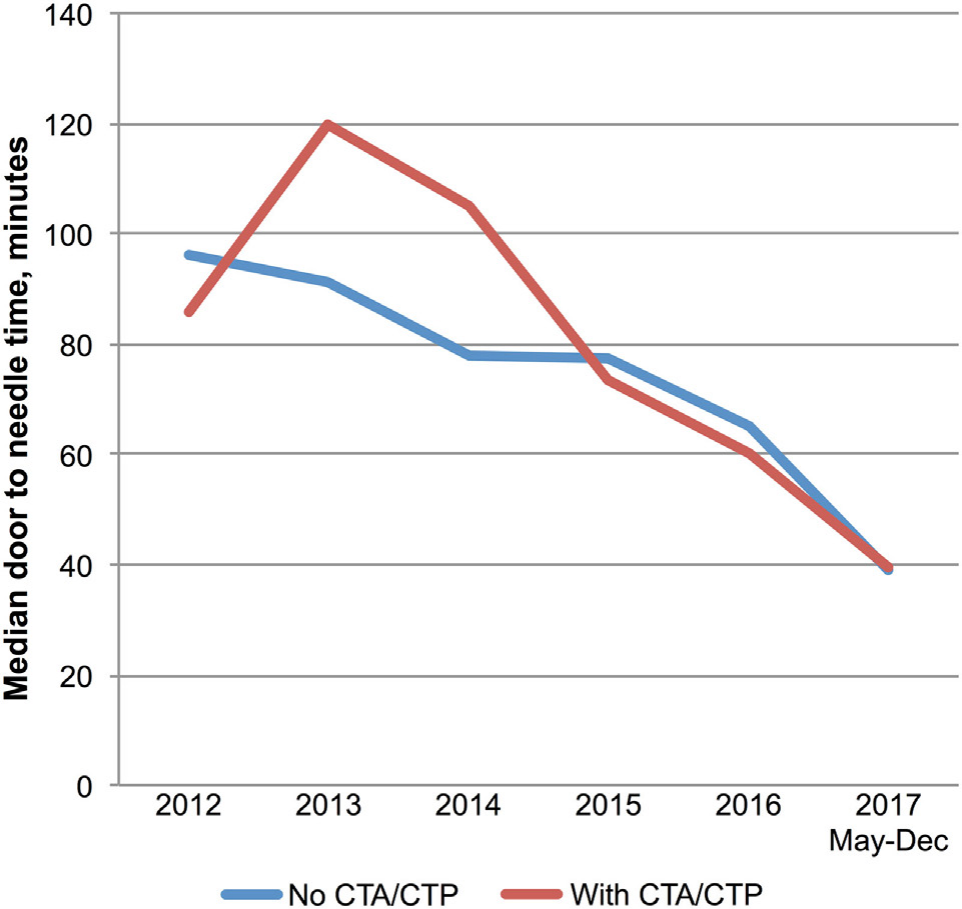
Multimodal imaging and treatment delays. Trends in door-to-needle time for patients treated with (*n* = 87) and without (*n* = 147) prior multimodal imaging. Abbreviations: CTA, computed tomography angiogram; CTP, computer tomography perfusion.

**Figure 4:**
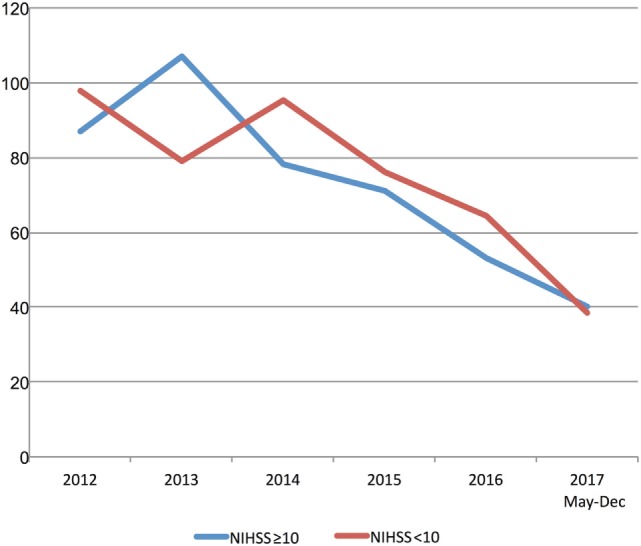
Stroke severity and treatment delays. Trends in door-to-needle time stratified by stroke severity. NIHSS, National Institutes of Stroke Scale Score.

Multimodal CT may have resulted in DNT delay in 2013 and 2014 based on a small number of patients with pre-treatment advanced imaging (Table 1; Figure 3). However, since 2015 pre-treatment multimodal CT did not result in a delay in DNT (Figure 3). More severe strokes (NIHSS ≥10) were initially associated with a faster DNT, but this difference diminished over time with no difference in treatment times in 2017 (Figure 4).

### Reduction in Other Time Metrics

**Figure 5:**
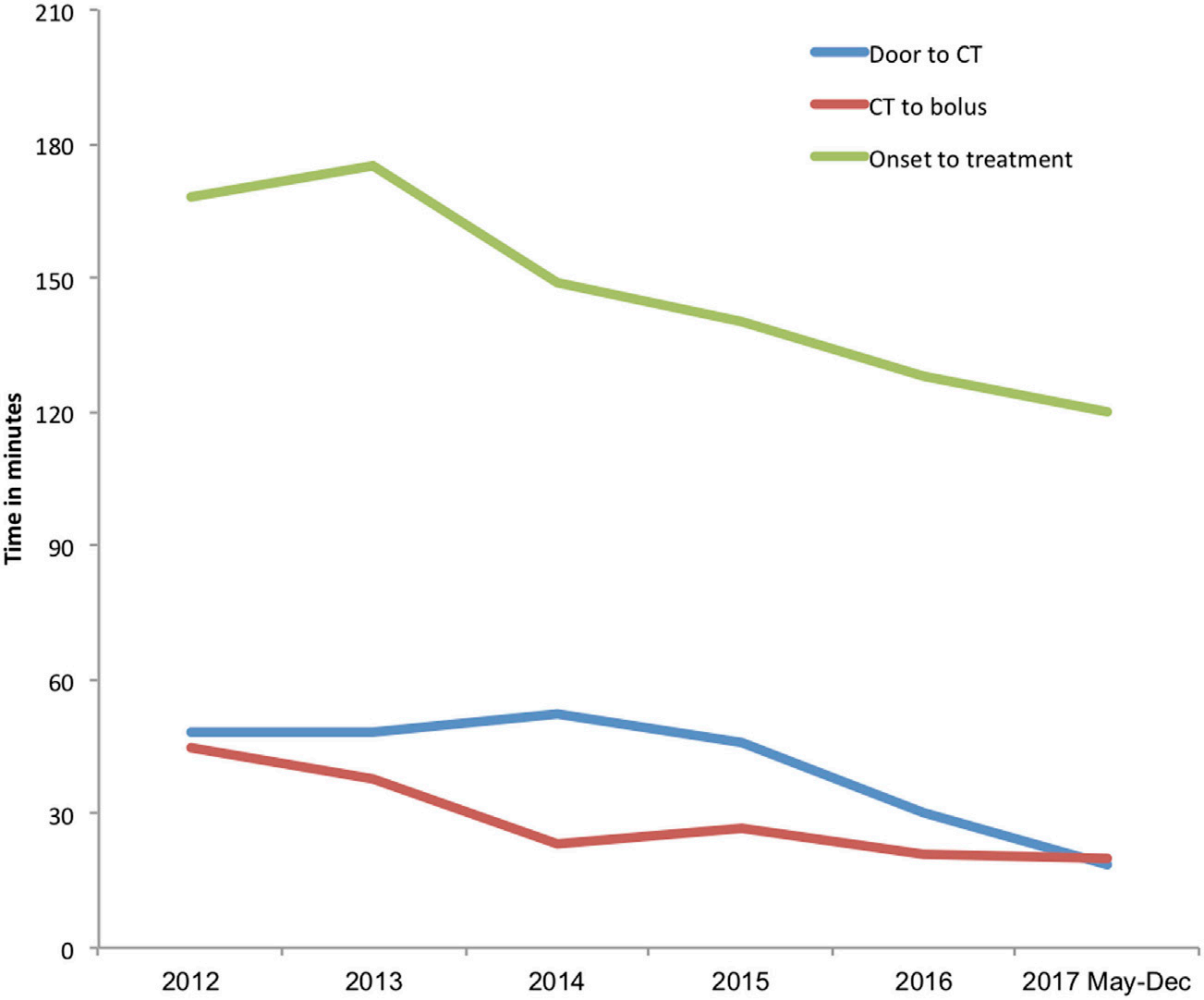
Trends in median door-to-imaging, imaging-to-treatment, and onset-to-treatment times. Annual median onset to treatment (green line), door-to-imaging (blue line), and imaging-to-treatment (red line) times in minutes.

There were also significant reductions in door-to-CT times and CT-to-needle times as shown in Table 3. In the final 8 months of 2017 after full implementation of the new stroke model the overall median door-to-CT time was 19 (11–24) minutes and the CT-to-needle time was 20 (14–31) minutes, representing reduction of 30 and 12 min, respectively compared with pre-intervention period (all *p*-values <0.01). The overall median onset to treatment time was also reduced from 168 (145–195) minutes in the pre-interventional period to 120 (87–155) minutes (*p* < 0.01) in 2017 (Figure 5; Table 3).

## Discussion

We have demonstrated that the key components of the Helsinki stroke thrombolysis model are transferrable with “real-word” resources and reduced median in-hours DNT from 87 to 34 min. The reduced stroke treatment delay was achieved despite increasing routine use of advanced imaging and administration of idarucizumab to a significant minority of thrombolysed patients on dabigatran. Similar significant reductions in DNT were made after-hours despite leaving running of the “Code Stroke” mostly to non-neurology medical residents.

Previous studies have already reported significant reductions in stroke DNT by having an organized thrombolysis pathway (3). Adaptation of the Helsinki stroke model to the Royal Melbourne Hospital in Australia resulted in an 18-min reduction in the in-hour DNT time in just 4 months (7). In the United States, the implementation of the American Heart Association/American Stroke Association national quality improvement initiative Target: Stroke—resulted in an increase in the proportion of tPA initiated within 60 min of hospital arrival from 30 to 53% in 1030 Get With the Guidelines—Stroke hospitals (8, 9). The 10 key best practice key strategies in the Target: Stroke initiative share important similarities with the Helsinki Stroke model with emphasis on hospital pre-notification, rapid imaging acquisition, clinical assessment, and tPA administration (6, 8). In other single centers in Europe, Canada, and the United States, DNT reductions of up to 63 min have been reported by adopting similar systematic acute stroke thrombolysis pathway (4, 13–17).

There were also significant reductions in other measured time metrics, with reductions in door-to-CT and CT-to-needle times by 61% and 38%, respectively. The overall onset-to-needle time also reduced to an overall median of 120 (87–155) minutes from 168 (145–195) minutes similar to reported else where (6, 7, 9, 14). It is possible that the 30-min reduction in door-to-CT time may have enabled treatment of additional patients presenting late in the treatment window, however, it is clear that the increased number of patients treated over the study period is not attributable to late time-window treatments as the onset to treatment time has decreased. The increased enthusiasm for stroke intervention with endovascular clot retrieval (18) and recent “FAST” public awareness campaigns by the Stroke Foundation of New Zealand are likely to have contributed to increased presentation of treatment eligible patients.

The main difference between our results and those published is the availability of on-site neurology residents. Most these centers have reported dedicated 24/7 (4–6, 13–17) on-site specialty neurology/stroke team in contrast to the set up at our hospital, where neurology on-site resident support is limited mainly to business hours. Although an on-site resident is available 24/7, the after-hours medical resident is additionally responsible for managing nephrology and infectious diseases patients in addition to acute neurology duties. Our setup is similar to other New Zealand centers where in 2015–2016 the median DNT was 70 (52–97) minutes and the median onset-to-needle time was 150 (119–195) minutes (19).

The use of advanced imaging prior to treatment resulted in a 20-min delay to treatment times in Helsinki (6). However, based on our experience, advanced imaging did not result in an additional delay to treatment. This is likely due to practice effect similar to the reported experience in Melbourne (7). Although giving tPA bolus after non-contrast imaging prior to advanced imaging would reduce DNT further, advanced imaging provides the on-call neurologists with diagnostic confidence particularly in patients presenting after hours. Our stroke mimic treatment rate (1.0%) in patients with pre-treatment multimodal CT was comparable to that reported in Helsinki (1.4%) and Melbourne (2.0%), where patients are generally reviewed physically by expert stroke neurologists (6, 7).

An important component of this model is regular education of non-neurology residents and St. John Ambulance paramedics. Regular formal 4-monthly feedback education sessions have been provided to rotating medical residents in addition to annual paramedic update on the Stroke pathway. Additional individual resident feedback sessions were also available throughout the year. In a study by Ruff et al. a formal case-based clinical “boot camp” for incoming junior neurology residents resulted in 25 min DNT reduction in patients managed by junior staff (20). Other centers have also incorporated regular feedback and education sessions as an ongoing feature of the stroke pathway (5, 14, 21). Our emphasis to the rotating residents is to contact the on-call neurologist early for all cases particularly in cases with diagnostic uncertainty adopting the “if in doubt, call out” mantra.

International best practice guidelines (12, 22) have recommended DNT of less than 60 min, with the Canadian guidelines recommending a 30-min median DNT target (22). Although our current results do not meet the 30-min DNT target, it is likely that with further practice, increasing familiarity and confidence with the pathway the DNT will be reduced further. We additionally anticipate DNT would be reduced by at least 5 min with the planned relocation of the CT suite from the first floor to the ED toward the end of 2018. This is likely also to reduce any delays associated with retrieving idarucizumab from the ED drug room for dabigatran-treated patients. Additionally our after-hours DNT was negatively influenced by non-operation of the stroke model overnight and the call-back roster system for CT radiographers. The after-hours DNT is likely to reduce further when immediate CT access is 24/7.

The main strength of this study is that our center is a general hospital, and acute stroke is managed by general ED, radiology, and neurology departments, without specialized stroke staff. Our results suggest that the key elements of thrombolysis models, such as the Helsinki Stroke model are transferrable to health settings with limited or “real world” resources. The main limitation of our data is that they are acquired from a single center: actual results from similar hospitals may vary according to local circumstances. Nonetheless, our results may actually under-estimate the impact of the new stroke model on DNT as the DNT is likely to reduce further with practice and the relocation of the CT suite. One further limitation is that we do not have long-term outcome data, so the impact of shorter DNT on patient outcome cannot be determined. However, it is likely that the patient outcomes would replicate that reported in literature (1).

In conclusion, we have demonstrated the transferrability of the Helsinki Stroke thrombolysis model to a large volume general hospital, reducing the median in-hours DNT to 34 min. Our results should encourage other thrombolysis capable hospitals to consider implementing similar stroke models to reduce stroke treatment delay and improve patient outcome.

## Ethics Statement

This retrospective study has approval from the national Health Disability Ethics Committee. As it is part of a routine observational quality registry with no patient contact or deviation from standard treatment, patient consent was not required.

## Author Contributions

TW designed the study, collected data, performed statistical analysis, and drafted the manuscript. JF, EC, SW, and MG collected data, reviewed, and edited the manuscript. AM was involved in study design, editing, and revision of the manuscript. All other authors reviewed and edited the manuscript for its intellectual content.

## Conflict of Interest Statement

AM has consulted for and received honoraria for talks from Boehringer Ingelheim, manufacturer of alteplase, and Stryker, manufacturer of endovascular devices for ischemic stroke. All other authors declare no conflict of interest.
